# Socioeconomic status determines sex-dependent survival of human offspring

**DOI:** 10.1093/emph/eot002

**Published:** 2013-03-01

**Authors:** David van Bodegom, Maarten P. Rozing, Linda May, Hans J. Meij, Fleur Thomése, Bas J. Zwaan, Rudi G. J. Westendorp

**Affiliations:** ^1^Department of Gerontology and Geriatrics, Leiden University Medical Center, PO Box 9600, 2300 RC Leiden; ^2^Leyden Academy on Vitality and Ageing, Poortgebouw LUMC, Rijnsburgerweg 10, 2333 AA Leiden; ^3^Department of Parasitology, Leiden University Medical Center, PO Box 9600, 2300 RC Leiden; ^4^Amphia Hospital, Postbus 90157, 4800 RL Breda; ^5^Department of Sociology, VU University Amsterdam, De Boelelaan 1081, 1081HV Amsterdam; ^6^Laboratory of Genetics, Wageningen University, PO Box 309, 6700 AH Wageningen and ^7^Institute of Biology, Leiden University, PO Box 9505, 2300 RA Leiden, The Netherlands.

**Keywords:** sex differences, Trivers–Willard, reproduction, offspring survival, offspring weight, Africa

## Abstract

**Background and objectives:** In polygynous societies, rich men have many offspring through the marriage of multiple wives. Evolutionary, rich households would therefore benefit more from sons, and according to the Trivers–Willard hypothesis, parents invest more in offspring of the sex that has the best reproductive prospects. We determined the sex differences in number of offspring, sex ratio of offspring, offspring survival and offspring weight in rich and poor households in a polygynous population.

**Methodology:** We studied a population of 28 994 individuals in Northern Ghana during an 8-year prospective follow-up. We determined the fertility rate for both men and women, sex ratio of 3511 newborn offspring and offspring survival in 16 632 offspring up to reproductive age (≤18 years). Also, we collected 9842 weight measurements of 1470 offspring up to the age of 3 years from growth charts of local clinics.

**Results:** In rich households, men have a lifetime number of 6.0 offspring, while for women this was 3.1. In line with evolutionary predictions, the male:female sex ratio was higher in rich households (0.52; poor households 0.49), sons had lower mortality in rich households (hazard ratio male versus female 1.06, *P* = 0.64; poor households: hazard ratio male versus female 1.46, *P* = 0.01) and sons also had higher weights in rich households (*P* = 0.008).

**Conclusions and implications:** In rich households, men have higher reproductive prospects in this polygynous society and, in line with Trivers–Willard, we registered more sons in rich households, sons had lower mortality and higher weights, maximizing the reproductive output in this society.

## Abstract (precis)

In a polygynous African society, rich men have much more life time offspring than women through the marriage of multiple wives. In line with evolutionary predictions, we found that in rich households more newborn sons were registered, sons had lower mortality and higher body weight. Conscious or unconscious, this maximizes reproductive output.

## BACKGROUND AND OBJECTIVES

In polygynous societies, richer men can afford to marry multiple wives and consequently increase their reproductive success. In terms of Darwinian fitness, rich households would therefore benefit more from sons with their higher reproductive prospects. According to the Trivers–Willard hypothesis, parents invest more in offspring of the sex that has the best reproductive prospects [1].

Although Trivers–Willard effects have been found in many animals, they are highly debated in humans. In a recent review of 422 studies in mammals, which investigated sex ratios at birth, excluding humans, a Trivers–Willard effect was consistently found in several species, while in other species, including non-human primates, more contradictory findings are found [2]. An important consideration here is that many human studies were performed in monogamous populations [3–5]. Here, large effects are not expected since in a monogamous society, there will mostly not be large differences in reproductive output of sons and daughters. In polygynous societies, however, a subset of more successful sons can have large reproductive output through the marriage of multiple wives. Previous studies that have examined sex ratios and the Trivers–Willard effect in polygynous human populations found no sex-specific survival differences dependent on status among the Bari of South America, nor among the Gabbra and Kipsigis of Kenya [6–8].

We studied reproductive output of men and women in poor and rich households in a large population of 28 994 individuals in a rural African society in the Upper East Region of Ghana with a high degree of polygyny. Second, we investigated the differences in offspring sex ratio, sex differences in offspring survival and offspring weight in poor and rich households.

## METHODOLOGY

### Study area

This study was conducted in the Garu-Tempane district in the Upper East region of Ghana. General fertility and mortality patterns have been described elsewhere [9]. The characteristics of the study population are presented in Table 1. The people are patriarchal, patrilineal and patrilocal and live in extended families, of which 48% are polygynous. During 8 years of follow-up from 2002 to 2010, we assessed reproduction and survival among 28 994 participants. The area is currently undergoing an epidemiological transition [10]. Drinking water was assessed on household level, water from boreholes was considered safe drinking water and water drawn from either open wells or from rivers was considered unsafe drinking water [11].

**Table 1.** Characteristics of the study population

Participants (n)	28 994
Male (%)	46
Female (%)	54
Tribe	
Bimoba (%)	66
Kusasi (%)	26
Other (%)	8
Households (n)	1703
Polygynous households (%)	48
Mean value of household possessions in US$ (mean (SD))	1063 (1021)
Safe drinking water (%)	80
*Number offspring*	
Numbers of offspring registered 2002–2010 (n)	3645
SES available (n)	3511
Offspring survival	
Offspring ≤ 18 years (n)	16 632
Follow-up (calenderyears)	2002–2010
Person years (n)	91 256
Mean follow-up (years)	5.5
Deaths during follow-up (n)	471
Weights of offspring	
Offspring ≤ 3 years with growth chart (n)	1470
Weight measurements (n)	9842
Average number of measurements per child (n)	7

### Socioeconomic status

In 2007, we designed a DHS-type questionnaire to assess the socioeconomic status (SES) of the households of the study participants using a free listing technique whereby we asked people from different villages of the research area, both male and female, in focus group discussions to list the household items of most value [12]. These self-listed property questionnaires are reported to be highly correlated to longer property questionnaires [13]. The resulting list of valuable items was comparable to part of the core welfare indications questionnaire from the World Bank and to the extended DHS asset list, adapted to our region. The list included different items, including mainly domestic livestock and different valuable household items comprising motorbikes, bicycles and iron roofing. The average wealth of the household possessions in market value of 2007 was 1063 US dollar with a SD of 1021 US dollar. The distribution was skewed to the right.

From these assets, a DHS wealth index was calculated. This was done as explained in paragraph 2.2 of the DHS wealth index comparative report [14]. Using SPSS factor analysis, the indicator variables were first standardized by calculating *z*-scores. Second, the factor coefficient scores or factor loadings are calculated. The DHS wealth index is the sum of the indicator values multiplied by the loadings. This index is itself a standardized score with a mean of 0 and a SD of 1. We defined poor and rich as the poorest 50% of households and the richest 50% of households divided by the median of the DHS wealth index.

### Fertility

From the registered newborn offspring and the observed person-years of fertile men and women during our 8-year follow up, we calculated the age-specific fertility rates. Next, we multiplied the age-specific fertility rates with the fraction of surviving men and women of these ages to calculate the number of offspring of each age group per year. The lifetime number of offspring was calculated as the sum of these numbers of offspring per year for all age groups multiplied by 5 since all age groups are 5-year age groups.

### Survival

The survival analysis used a multivariable left-truncated Cox regression analysis adjusted for sex, tribe and drinking source. We found no evidence that the assumption of proportionality of hazards was violated. The left-truncated plots represent age-specific survival probabilities calculated from the 8-year follow-up rather than a prospective lifetime follow-up. For the survival analysis up to reproductive age, we included all offspring up to 18 years. This survival analysis was performed on all person-years observed ≤18 years during our 8-year follow-up. Some individuals were followed 8 years below the age of 18 years; some individuals were followed both below age 18 years and above and in those cases only the person-years observed below age 18 years were included in the analysis. In total, we followed 16 632 individuals for 91 256 person-years which makes an average of 5.5 years follow-up below the age of 18 years per individual observed. During our follow-up, we observed 471 deaths below the age of 18 years.

### Weights

The weights of the offspring were obtained from growth charts of local health clinics in 2008. The clinics use hanging scales to measure the weight and use growth charts from the Ghana Health Service, adapted from the World Health Organization. For the separate sexes of each age, we standardized the weights on age and sex by calculating SDS or *z*-scores by subtracting the mean from the observed weight and dividing by the standard deviation.

On average, we had seven measurements per child during their first 3 years of life. To take these repeated measures into account and not treat them as independent measures, we used a linear mixed model. In the model, we adjusted/corrected for tribe of the offspring. The offspring from different tribes in the area have very different biometrics. Some tribes have cows, and the offspring of these tribes drink milk. Therefore, these offspring have less stunted growth and also do not suffer from (protein) malnutrition. We also adjusted the model for drinking source and the month and year of measurement, as weights fluctuated dependent on the season and year (Supplementary Fig. S1). The point estimates presented in this article are estimates derived from this model and therefore do not always add up to zero for each age.

### Ethics

Ethical approval was given by the Ethical Review Committee of the Ghana Health Service, the Medical Ethical Committee of the Leiden University Medical Centre in Leiden, The Netherlands, and by the local chiefs and elders of the research area. 

All analyses were performed with Stata 11.0 (StataCorp LP, TX, USA).

## RESULTS

**Figure 1.**
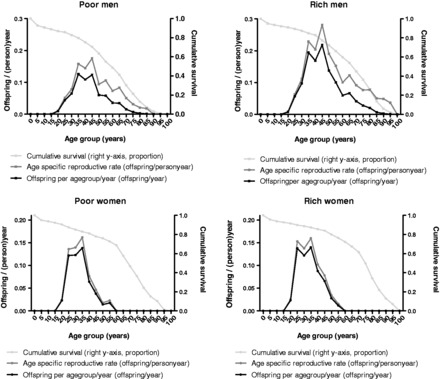
Cumulative survival, age-specific fertility rate and offspring per year for poor and rich men and women of different age groups.

We visited the research area annually from 2002 to 2010. Each year we registered the deaths, migration and newborn offspring. Figure 1 shows the cumulative survival, age-specific fertility rates and number of offspring for men and women of different age groups from poor and rich households.

**Figure 2.**
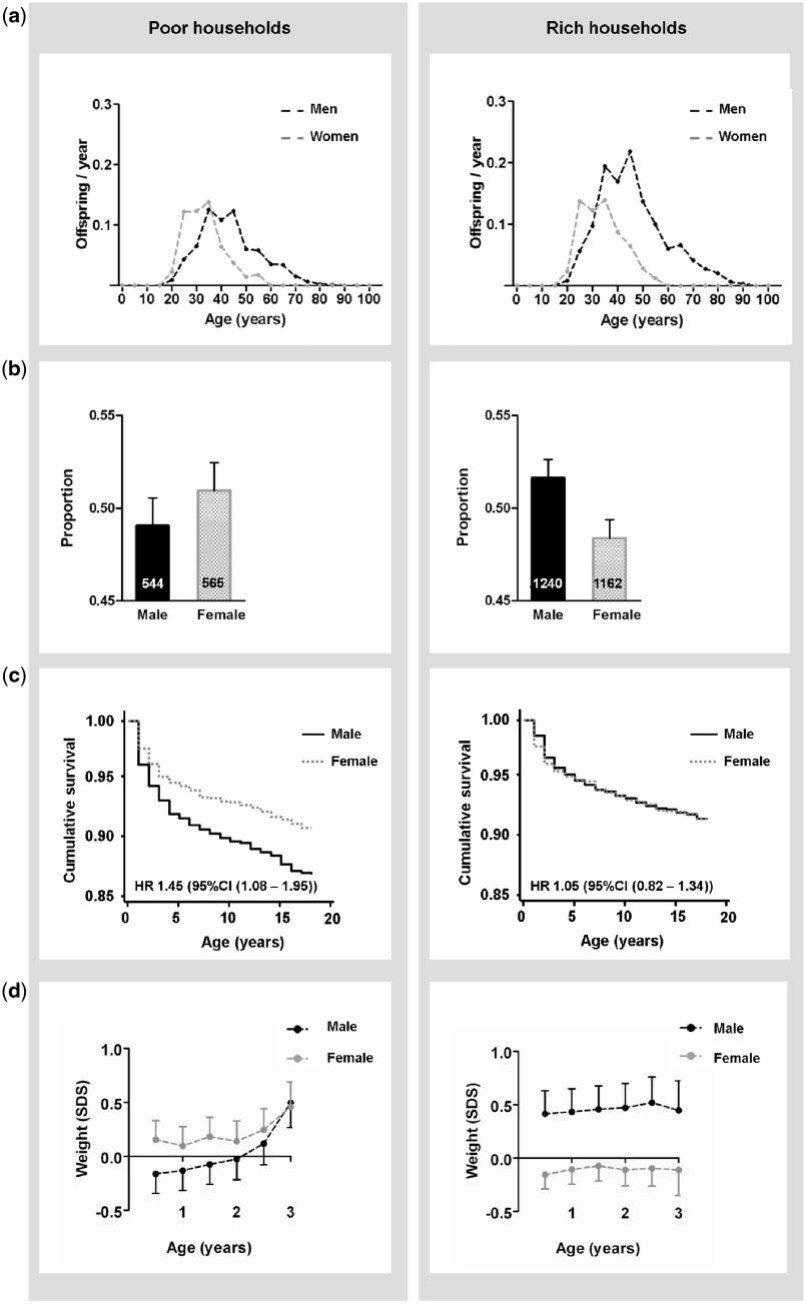
Offspring per year (**a**), sex of offspring (**b**), offspring survival (**c**) and offspring weight (**d**) in poor and rich households. Error bars indicate standard errors. SDS = Standard Deviation Score.

Figure 2a compares these numbers of offspring born to fathers and mothers of different ages in poor and rich households. The people in the research area are polygynous and the man must pay a bride price of four cows to arrange a marriage. Consequently, richer men are able to increase their number of wives and hence offspring. Taking the age-specific fertility rate and survival to these ages into account, in poor households the total number of lifetime offspring, represented by the area under the curve in the figure, was 3.4 offspring for men and 2.7 offspring for women. In rich households, the total number of lifetime offspring was 6.0 offspring for men, whereas it was 3.1 offspring for women.

Studies have shown a strong heritability of SES in pre-transitional societies [15]. This seems applicable to this population also, since income is generated largely through agriculture and sons inherit the cattle and land of their fathers. If offspring inherits the SES from their parents and rich men have better reproductive prospects, one could hypothesize that rich households would benefit more from sons, which would create an opportunity for selection on sex-specific survival dependent on SES. We compared the sex ratio of offspring, offspring survival and offspring weights in poor and rich households.

Figure 2b shows the sex ratio of the registered offspring in the research area. Of all 3685 offspring, we had socioeconomic information on 3511 offspring. In poor households, we registered 544 male offspring and 565 female offspring (male:female sex ratio 0.49). In rich households, we registered 1240 male offspring and 1162 female offspring (male:female sex ratio 0.52). Since we did not register the offspring at birth, but during the annual field visit, these sex ratios are secondary sex ratios at an average age of 6 months.

Second, we studied survival of 16 632 offspring up to reproductive age (≤18 years) (Fig. 2c). In poor households, sons had much higher mortality risk compared with daughters (hazard ratio (HR) 1.46 [95% CI 1.08–1.96]; *P* = 0.01). In rich households, however, mortality risk of sons was similar to that of daughters (HR 1.06 [95% CI 0.84–1.33]; *P* = 0.64, *P* for interaction = 0.09).

**Figure 3.**
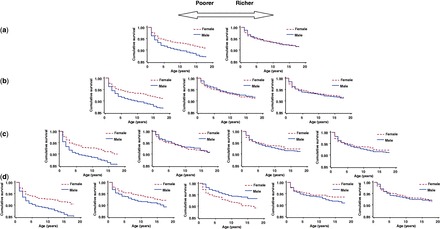
Offspring survival ≤18 years dependent on socioeconomic status (SES) in different strata of wealth (DHS wealth index). (**a**) Split by median (poor versus rich), (**b**) tertiles of SES, (**c**) quartiles of SES and (**d**) quintiles of SES.

To further investigate the observed sex differences, we also looked at the survival differences in different strata of SES. Figure 3 shows the survival differences for male and female offspring stratified in different strata of SES. The accompanying HRs are reported in Table 2. These analyses show that the sex-specific survival differences dependent on SES are largely due to a reduced survival of male offspring in the poorest households.

**Table 2.** Hazard ratios for mortality ≤18 years (male versus female)

	HR	95% CI	*P*
Poorest 50%	1.46	(1.08–1.96)	**0.01**
Richest 50%	1.05	(0.84–1.33)	0.64
			
First tertile	1.51	(1.11–2.05)	**0.008**
Second tertile	0.96	(0.70–1.33)	0.81
Third tertile	1.15	(0.83–1.59)	0.4
			
First quartile	1.43	(1.03–2.00)	**0.03**
Second quartile	0.97	(0.67–1.41)	0.89
Third quartile	1.24	(0.86–1.81)	0.25
Fourth quartile	1.11	(0.75–1.65)	0.59
			
First quintile	1.58	(1.09–2.29)	**0.02**
Second quintile	1.35	(0.88–2.07)	0.17
Third quintile	0.68	(0.45–1.03)	0.07
Fourth quintile	1.44	(0.92–2.25)	0.11
Fifth quintile	1.09	(0.72–1.76)	0.68

Bold values indicate significance at p < 0.05

Third, we analyzed the weights of offspring using repeated measurements from growth charts of the local health clinics. In an analysis of 9842 age and sex standardized measurements among 1470 offspring up to the age of 3 years, daughters had higher weights in poor households, whereas sons had higher weights in rich households (Fig. 2d). These differences in sex-specific weight gain were significantly different (*P* for interaction = 0.008).

## CONCLUSIONS AND IMPLICATIONS

We observed sex-specific effects of SES on the sex ratio of offspring, offspring survival and offspring weight. Several points should be discussed when interpreting these results.

First, concerning the high ages of continued reproduction in this area. Since there is no official registration of births in this area, the ages are estimated ages by three independent observers, both local and Dutch fieldworkers. We used all information available to come to a best estimate, most notably the relation to other family members with known ages, but some ages could be estimated too low and some too high. Although we did our best to come to an objective estimate, old age carries a certain status in this area, and it is possible that more ages are overestimations than underestimations. This could explain the unusual high age of retained fertility for some women and it is also possible that the high reproductive output of some old men could be a little less extreme. Although misclassification of ages does not change the interaction of wealth and sex as we describe in this article, it is important to recognize this when interpreting the fertility data.

Second, the sex ratios are sex ratios during registration at our annual field visit. Therefore, they are secondary sex ratios at an average age of 6 months and they do not necessarily reflect sex ratios at birth. Therefore, they could be the result of early mortality differences instead. We have observed mortality differences up to 18 years and it is expected that these differences also exist in the first 6 months of life.

Another important point to discuss in this polygynous society is that men that fail to marry migrate to the south of Ghana to work in poor conditions in large cities or large-scale agricultural plantations. We have no estimate of their reproductive output but it is possible that this is low. Since the men that migrate are preferential poor males, the fertility figures for poor males in the research area are most probably overestimations of the reproductive output of all men born in poor households in the area. The contrast in reproduction between poor and rich is therefore probably even stronger than presented here. It is even possible that the lifetime number of offspring of poor women is greater than the lifetime number of offspring of poor men if this would be taken into account. However, it is not possible to calculate this without knowing the exact fertility characteristics of the men that migrate. This does not change our conclusions, however, and in fact, it is possible that the Trivers–Willard effect could even be stronger than presented here.

Concerning the mechanism behind the observed sex differences dependent on SES, two possible explanations exist. First, they could be a reflection of higher intrinsic vulnerability of sons to poor conditions. Looking at the mortality patterns in poor and rich households, Fig. 3 shows that the differences are largely determined by a higher mortality of sons in poor conditions. It is known that men have higher mortality risks throughout life in almost all countries and in this harsh environment, this could be the principle mechanism behind the observed survival differences dependent on SES [16]. Second, our observations are also in line with differences in parental investment as hypothesized by Trivers and Willard. The observed sex differences in weight could reflect differences in parental nursing habits; sex differences in breastfeeding have previously been observed in Poland and the Caribbean [17, 18]. These differences in parental behavior do not have to be based on conscious decisions. Previous studies among the Mukogodo of Kenya also showed that in a male-centered society, parental behavior can, maybe not even always consciously, be female oriented in a society where all Mukogodo are poor in relation to the Masaai [19]. We do not have observations on parental behavior in our study. Although this would be interesting, from an evolutionary perspective not the mechanism but the number of surviving male and female offspring is most relevant.

A last thing to consider is a potential effect that birth order could have on the observed patterns. It could be expected that the first-born son would be preferred; because he would inherit the wealth and therefore have high reproductive prospects while later born sons would be less favored. Unfortunately, we do not have reliable data on this, but we are planning to collect this in the future. On the other side, although the oldest son inherits the house, his brothers together with their wive(s) will often live with him in his household. Also, it is important to realize that in this society, possessions are not owned individually but are shared to a high degree among the (male) kin of the household.

Whether the sex differences that we have observed in our study reflect the higher vulnerability of sons to poor conditions, or reflect a sex-specific parental investment as proposed by Trivers and Willard, the net result is the same; sons are better off in richer households which maximizes the reproductive prospects of households in this polygynous society. In fact, the two explanations are not mutually exclusive. The Trivers–Willard hypothesis refers to an ultimate explanation in terms of evolutionary optimization. Differential vulnerability to poor conditions is a proximate explanation referring to a potential mechanism, even if unspecified.

## SUPPLEMENTARY DATA

Supplementary data are available at *EMPH* online.

## FUNDING

This research was supported by the Netherlands Foundation for the advancements of Tropical Research (WOTRO); the Netherlands Organization for Scientific Research (NWO); the EU-funded Network of Excellence LifeSpan, an unrestricted grant of the Board of the Leiden University Medical Center and the Association Dioraphte. None of these organizations had any role in the design, analysis, interpretation or report of the study.

**Conflict of interest**: none declared.

Funding to pay the Open Access publication charges for this article was provided by the Leyden Academy on Vitality & Ageing.

## Supplementary Material

Supplementary Data

## References

[1] Trivers RL, Willard DE (1973). Natural selection of parental ability to vary the sex ratio of offspring. Science.

[2] Cameron EZ (2004). Facultative adjustment of mammalian sex ratios in support of the Trivers–Willard hypothesis: evidence for a mechanism. Proc Roy Soc Lond B.

[3] Cameron EZ, Dalerum FA (2009). Trivers–Willard effect in contemporary humans: male-biased sex ratios among billionaires. PLoS One.

[4] Chacon-Puignau GC, Jaffe K (1996). Sex ratio at birth deviations in modern Venezuela: the Trivers–Willard effect. Soc Biol.

[5] Gaulin SJ, Robbins CJ (1991). Trivers–Willard effect in contemporary North American society. Am J Phys Anthropol.

[6] Zaldivar ME, Lizarralde R, Beckerman S (1991). Unbiased sex ratios among the Bari: an evolutionary interpretation. Hum Ecol.

[7] Mace R (1996). Biased parental investment and reproductive success in Gabbra pastoralists. Behav Ecol Sociobiol.

[8] Borgerhoff Mulder M (1998). Brothers and sisters. How sibling interactions affect optimal parental allocations. Hum Nat.

[9] Meij JJ, van Bodegom D, Ziem JB (2009). Quality–quantity trade-off of human offspring under adverse environmental conditions. J Evol Biol.

[10] Meij JJ, de Craen AJ, Agana J (2009). Low-cost interventions accelerate epidemiological transition in Upper East Ghana. Trans R Soc Trop Med Hyg.

[11] Kuningas M, May L, Tamm R (2009). Selection for genetic variation inducing pro-inflammatory responses under adverse environmental conditions in a Ghanaian population. PLoS One.

[12] van Bodegom D, May L, Kuningas M (2009). Socio-economic status by rapid appraisal is highly correlated with mortality risks in rural Africa. Trans R Soc Trop Med Hyg.

[13] Morris SS, Carletto C, Hoddinott J (2000). Validity of rapid estimates of household wealth and income for health surveys in rural Africa. J Epidemiol Commun Health.

[14] Rutstein SO, Johnson K (2004). The DHS Wealth Index. DHS Comparative reports No. 6.

[15] Borgerhoff Mulder M, Bowles S, Hertz T (2009). Intergenerational wealth transmission and the dynamics of inequality in small-scale societies. Science.

[16] Kalben B (2000). Why men die younger: causes of mortality differences by sex. North Am Actuarial J.

[17] Koziel S, Ulijaszek SJ (2001). Waiting for Trivers and Willard: do the rich really favor sons?. Am J Phys Anthropol.

[18] Quinlan RJ, Quinlan MB, Flinn MV (2008). Local resource enhancement and sex-biased breastfeeding in a Caribbean community. Curr Anthropol.

[19] Cronk L (1991). Intention versus behaviour in parental sex preferences among the Mukogodo of Kenya. Biosoc Sci.

