# Supplementary material for: Socioeconomic status determines sex-dependent survival of human offspring
Source: Evol Med Public Health. 2013 Mar 1;2013(1):37–45. doi: 10.1093/emph/eot002 (PMC3868360; doi:10.1093/emph/eot002)
Supplement: Supplementary Data [file supp_2013_1_37__index.html]

Socioeconomic status determines sex-dependent survival of human offspring — Supplementary Data 

# Socioeconomic status determines sex-dependent survival of human offspring

## Supplementary Data

files

**Files in this Data Supplement:**

- Supplementary Data - docx file
