# Supplementary material for: Socioeconomic status determines sex-dependent survival of human offspring
Source: Evol Med Public Health. 2013 Mar 1;2013(1):37–45. doi: 10.1093/emph/eot002 (PMC3868360; doi:10.1093/emph/eot002)

Supplementary figure S1. Offspring weights of offspring ≤ 3 years dependent on the season. Estimates indicate the estimates from a linear mixed model modeling the Z-score (SDS score) of the calendar month of measurement corrected for age, sex, tribe, drinking source and year of measurement.


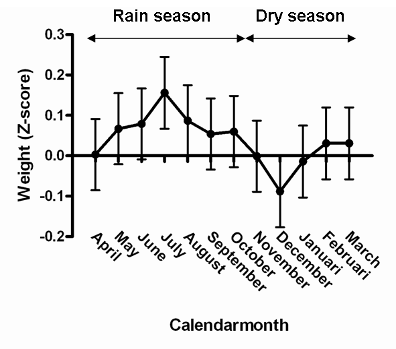

Supplement: Supplementary Data [file supp_eot002_Supplementary_figure_S1.docx]
